# Natural Co-Occurrence of Multiple Mycotoxins in Unprocessed Oats Grown in Ireland with Various Production Systems

**DOI:** 10.3390/toxins13030188

**Published:** 2021-03-04

**Authors:** Lorenzo De Colli, Karl De Ruyck, Mohamed F. Abdallah, John Finnan, Ewen Mullins, Steven Kildea, John Spink, Christopher Elliott, Martin Danaher

**Affiliations:** 1 Institute for Global Food Security, School of Biological Sciences, Queen’s University Belfast, Belfast BT9 5DL, UK; Chris.Elliott@qub.ac.uk; 2 Food Safety Department, Teagasc Food Research Centre, Ashtown, Dublin 15, Ireland; Karl.DeRuyck@teagasc.ie (K.D.R.); Martin.Danaher@teagasc.ie (M.D.); 3 Department of Food Technology, Faculty of Bioscience Engineering, Coupure Links 653, 9000 Gent, Belgium; Mohamed.Fathi@ugent.be; 4 Department of Forensic Medicine and Toxicology, Faculty of Veterinary Medicine, Assiut University, Assiut 71515, Egypt; 5 Crops Science Department, Teagasc, Oak Park, Carlow R93 XE12, Ireland; John.Finnan@teagasc.ie (J.F.); Ewen.Mullins@teagasc.ie (E.M.); Stephen.Kildea@teagasc.ie (S.K.); John.Spink@teagasc.ie (J.S.)

**Keywords:** mycotoxins, emerging, masked, oats, food safety, LC-MS/MS, Ireland

## Abstract

The natural co-occurrence of 42 mycotoxins was investigated in unprocessed oat grains grown in Ireland. The sample set included a total of 208 oat crops harvested during 2015–2016 and produced using conventional, organic, or gluten free farming systems. A range of different toxins was identified, including the major type A (neosolaniol, HT-2 and T-2 toxins, T-2 triol, and T-2-glucoside, co-occurring in 21 samples) and B trichothecenes (deoxynivalenol, nivalenol, and deoxynivalenol-3-glucoside), enniatins (B1, B, and A1, co-occurring in 12 samples), as well as beauvericin, alternariol, mycophenolic acid, and sterigmatocystin. The influences of sowing season, year, and production system were investigated, eventually indicating that the latter factor may have a higher impact than others on the production of certain mycotoxins in oats. The most frequently quantified compounds were HT-2 (51%) and T-2 (41%) toxins, with gluten free oats containing significantly lower concentrations of HT-2 compared to conventionally produced oats. Although the prevalence and concentrations of mycotoxin found in oat samples in this study should be substantially reduced by processing. However, as mycotoxin occurrence is clearly influenced by multiple factors, controlled field trials should be carried out to define optimal agronomic practices and mitigate mycotoxin production. Furthermore, this work highlights the need for regularly testing cereal-based foods with multi-residue analytical methods with wider specificities than the traditionally screened and regulated toxins, to generate knowledge on the occurrence of several mycotoxins that are, to date, rarely investigated.

## 1. Introduction

*Avena sativa*, commonly known as oats, is a cereal belonging to the *Poaceae* grass family that has traditionally been used as animal feed, particularly for horses and dairy cows. In recent years, the beneficial nutritional and physiological effects of oat products have generated an increase in nutrition-conscious consumer demand. The characteristic feature of oat grains is the favourable profile of amino acids, vitamins, minerals, and essential unsaturated fatty acids [1,2]. Food uses for oats include oat bran, oatmeal, and oat flakes, which are mainly consumed as breakfast cereals, and other products such as oat flour, nutritional bars, and food ingredients [3]. Oats account for less than 2% of the global cereal production, being ranked sixth in annual harvest weight after corn, wheat, barley, sorghum, and millet [4]. In Ireland, it is an economically important crop, representing the third most grown cereal [5]. Moreover, it is the second highest contributor to whole grain intake, providing 16%, 18%, and 26% of the whole grain intakes for children, teenagers, and adults, respectively. The mean daily whole grain intake was previously calculated to be 18.5 g/d for children and 23.2 g/d for teenagers, while for Irish adults 27.8 g/d were consumed [6,7].

However, oats, like other cereals, are susceptible to pathogenic fungal infestations. Several fungal species, mainly from the *Fusarium*, *Aspergillus*, *Alternaria* and *Penicillium* genera, are capable of attacking the crops in the field or the grains during the storage. Under certain environmental conditions, the fungal contamination results in the production of a plethora of toxic secondary metabolites known as mycotoxins. These metabolites can elicit a wide range of severe toxic effects in humans and animals that were exposed mainly via ingestion of contaminated food or feed. It has been estimated that up to 25% of worldwide food crops are contaminated by mycotoxins, but these figures may greatly underestimate their occurrence [8], resulting in significant economic losses and grave public health concerns. Consequently, the international authorities have established maximum permissible levels of certain mycotoxins in a variety of raw crops, animal feeds, and foods and beverages for human consumption, based on the available data regarding their toxic effect(s), occurrence and consumption patterns, as well as other economic, sociocultural, and political considerations. In Europe, the regulated mycotoxins currently include aflatoxins (AFB1, AFB2, AFG1, AFG2, and AFM1, deoxynivalenol (DON), fumonisins (FB1 and FB2), ochratoxin A (OTA), zearalenone (ZEN), patulin (PAT), and citrinin (CIT) [9]. In addition, maximum recommended levels exist for some mycotoxins, such as T-2 and HT-2 toxin [10]. However, several hundred more mycotoxins and fungal metabolites have been reported in the literature [11]. These compounds include the so called “emerging” and “masked” toxins, a group of less studied fungal secondary and plant-modified metabolites [12,13]. Due to the limited data currently available regarding their occurrence and toxicity, as well as the lack of specific legislation, the emerging and masked mycotoxins are not routinely screened by food testing laboratories. This gives rise to the possibility for consumer cereal products, including oats, to be contaminated by these unmonitored toxins.

In the last two decades, a number of surveys have been conducted in oats and other cereals at global level, particularly in Europe and North America [14,15,16,17,18,19,20,21,22,23,24,25,26,27,28,29,30,31,32,33,34]. Although some of these surveys reported the presence of certain emerging and masked mycotoxins, such as enniatins and/or the glucoside derivative of DON (D3G), the vast majority of reports included only the regulated mycotoxins. Specifically regarding oats grown in Ireland, limited data on mycotoxin contamination is available, with only the regulated mycotoxins having been investigated using relatively small sample sets [35,36]. The objective of this work was to investigate the natural co-occurrence of mycotoxins in raw oats produced with different production systems on the island of Ireland. The full list of mycotoxins assessed in this study includes: 3-acetyl-deoxynivalenol (3ADON), 15-acetyl-deoxynivalenol (15ADON), aflatoxin B1 (AFB1), aflatoxin B2 (AFB2), aflatoxin G1 (AFG1), aflatoxin G2 (AFG2), α-zearalenol (α-ZEL), alternariol (ALTOH), alternariol monomethyl ether (AME), andrastin A (ANDA), beauvericin (BEA), β-zearalenol (β-ZEL), deoxynivalenol (DON), deoxynivalenol-3-glucoside (D3G), diacetoxyscirpenol (DAS), enniatin A (ENA), enniatin A1 (ENA1), enniatin B (ENB), enniatin B1 (ENB1), fumonisin B1 (FB1), fumonisin B2 (FB2), fumonisin B3 (FB3), fusarenon-X (FUS-X), gliotoxin (GLIO), HT-2 toxin (HT-2), mycophenolic acid (MPA), neosolaniol (NEO), nivalenol (NIV), ochratoxin A (OTA), ochratoxin B (OTB), patulin (PAT), roquefortin C (ROQC), roquefortin E (ROQE), sterigmatocystin (STER), T-2 toxin, T-2 triol (T2-3OH), tentoxin (TEN), territrem B (TERRB), wortmannin (WORT) and zearalenone (ZEN).

## 2. Results

### 2.1. Overall Co-Occurrence of Mycotoxins

A total of 20 (out of 42) different mycotoxins were detected in the 208 samples tested from the years 2015 and 2016. The results of this analysis are summarised in Table 1, which shows the percentage and number of the positive detections, median, mean, minimum, and maximum values in the oat samples assayed. Out of the twenty mycotoxins found to be present in the unprocessed oats, fifteen were detected in both years of harvesting. The remaining mycotoxins (OTA, OTB, ALTOH, ANDA, and ZEN) were detected in 2015 samples only. A total of 54 samples (26%) did not contain mycotoxins at concentrations above the LOQs (Table 1). The most frequently detected toxins were HT-2 (51%) and T-2 (41%), followed by MPA (28%), T2-3OH (28%), and ENB (24%). The regulated toxins DON, OTA, and ZEN occurred in 2%, 4%, and 1%, respectively. None of these samples contained levels of DON and ZEN exceeding the current legislative limits [9], while the levels of OTA were above the limits in six samples.

**Figure 1:**
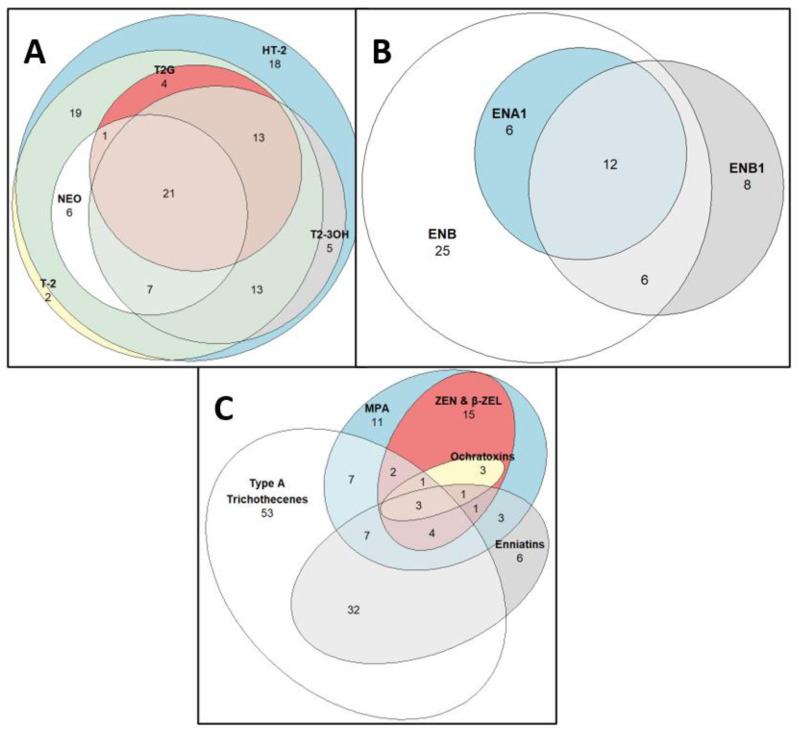
Euler diagram illustrating the major co-occurrence patterns (number of samples) of mycotoxins detected in unprocessed Irish oats collected in 2015–2016. (**A**) type A trichothecenes; (**B**) Enniatins; (**C**) Figure A + Figure B + zearalenone (ZEN), *β*-zearalenol (*β*-ZEL) and *Penicillium* toxins (mycophenolic acid (MPA), ochratoxin A (OTA) and ochratoxin B (OTB)).

A total of 127 out 208 samples were co-contaminated with two mycotoxins. An overview of the co-occurrence patterns of mycotoxins in contaminated samples is shown in Figure 1. In particular, T-2 and HT-2 co-occurred in 84 oat samples (40%), while 21 samples (10%) contained the five type A trichothecenes NEO, HT-2, T-2, T2-3OH, and T2G. The enniatins ENB, ENB1, and ENA1 co-occurred in 12 samples (6%), while 18 samples (9%) were co-contaminated with ENB and ENB1. All five type A trichothecenes as well as these three enniatins were found to co-contaminate 46 oat samples (22%). In addition, all the samples containing MPA were also found to contain ZEN, β-ZEL, OTA, and OTB. The mycotoxins that were not detected and their corresponding limit of quantification (LOQs) are listed in Supplementary Table S1.

### 2.2. Occurrence of Mycotoxins Based on the Sampling Year and Sowing Season

Evaluating the seasonal occurrence of mycotoxins, differences could be observed by comparing each season across two years, representing four individually defined growing conditions (i.e., spring 2015 vs. winter 2015 vs. spring 2016 vs. winter 2016, Figure 2). The enniatins and both trichothecenes of type A (NEO, HT-2, T-2, and their modified forms T2-3OH and T2G) and type B (DON and D3G) appeared to be more frequent in spring 2016 than spring 2015 oats. However, winter-sown oats showed the highest prevalence of HT-2 and T-2, particularly in regard to winter 2015, where their presence was detected in more than 50% of the samples. Similarly, ENB1 and ENA1 were more prevalent in winter oats, while ENB was more frequent in spring oats, both years. A similar trend was observed for MPA and β-ZEL, showing their highest and lowest prevalence in spring and winter of both years, respectively.

**Figure 3:**
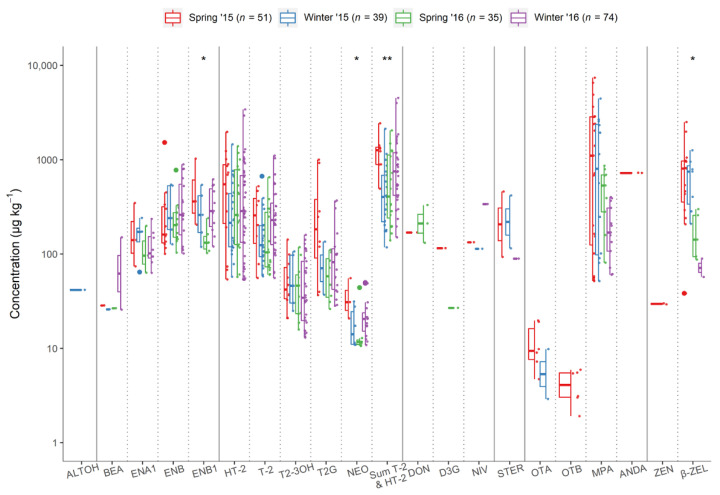
Concentrations (µg kg^−1^) of the mycotoxins detected in spring and winter oats over two consecutive years (2015–2016); * significant difference according to Kruskal–Wallis H test: (*) *p* < 0.05; (**) *p* < 0.01. Data beyond the ends of the whiskers are outlying points and are plotted individually.

The Kruskal–Wallis H test indicated that there were differences in the combined HT-2 and T-2 levels between seasons (Figure 3). Post hoc pairwise comparisons identified significantly different distributions between spring 2015 vs. winter 2015 (*p* = 0.007), winter 2015 vs. winter 2016 (*p* = 0.042), and spring 2015 vs. spring 2016 (*p* = 0.047). However, post hoc analysis did not confirm any significant differences between seasonal distributions of NEO, ENB1, nor β-ZEL, after correcting for multiple comparisons. For most mycotoxins that were detected in both sampling years, their concentration distributions were not found to differ significantly between the two sampling years (data not shown). The only exception was that β-ZEL was significantly higher (*p* < 0.001) in the samples from 2015. Furthermore, as shown in Figure 3, the regulated mycotoxins OTA and ZEN, along with OTB, ALTOH, and ANDA, were detected only in 2015 samples.

### 2.3. Occurrence of Mycotoxins in Unprocessed Irish Oat Samples Based on Production Practice

**Figure 4:**
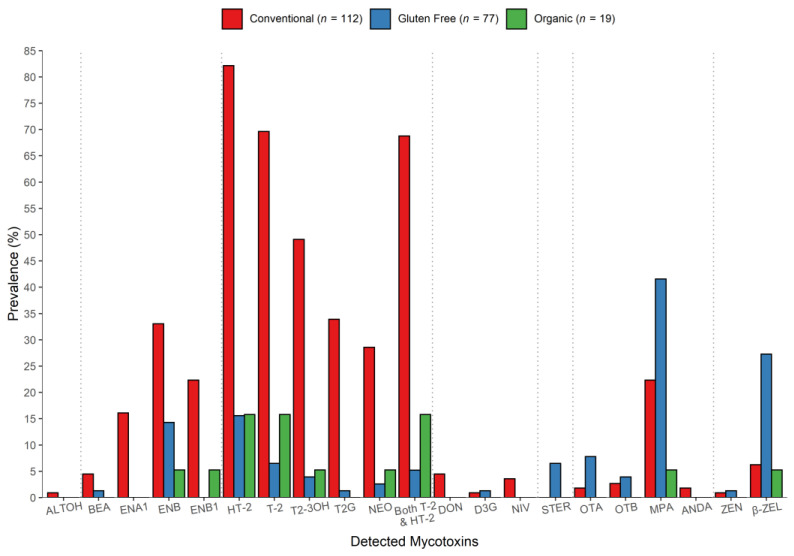
Prevalence (%) of the mycotoxins detected in oat samples from conventional, gluten free, and organic production systems collected over two years (2015–2016).

Type A trichothecenes, in particular HT-2, T-2 and their modified forms T2-3OH and T2G were more frequently detected in the conventional oats than either the “gluten free” or organic oats; as many as 82%, 70%, 50%, and 34%, respectively, of conventional samples were contaminated by detectable levels of each of these mycotoxins (Figure 4). Similarly, ENB and ENB1 were at least twice more prevalent in the conventional oats than in oats from the other two systems (ENB1 was not detected in gluten free oats), while ENA1 was detected only in the conventionally grown samples. Conversely, OTA, OTB, and STER occurred more frequently in the gluten free oats, although their prevalence did not exceed 8%, 4%, and 6%, respectively. DON was detected in conventional oats only, while D3G was not measured in organic oats. However, both of these toxins contaminated less than 5% of all samples.

**Figure 5:**
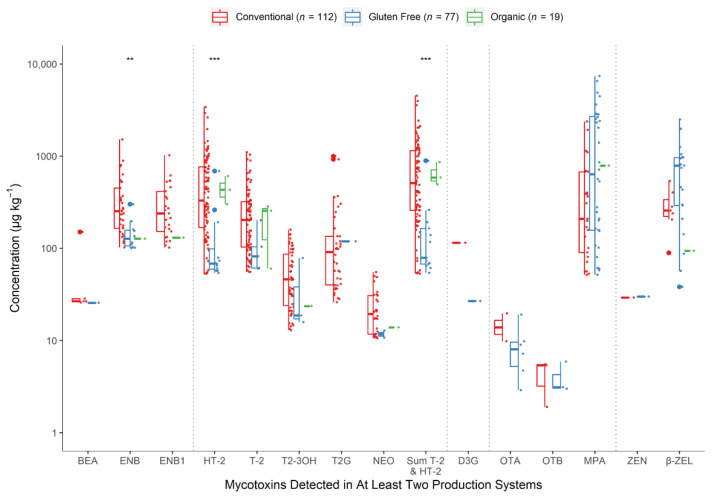
Differences in mycotoxin concentrations (µg kg^−1^) in oat samples from conventional, gluten free, and organic production systems collected over two years (2015–2016); * significant difference according to Kruskal–Wallis H test: (**) *p* < 0.01; (***) *p* < 0.001. Data beyond the ends of the whiskers are outlying points and are plotted individually.

A comparison of the mycotoxin levels measured in oats produced by conventional, gluten free, or organic systems is shown in Figure 5. Post hoc pairwise analyses identified samples from the gluten free farming system as containing significantly lower concentrations of HT-2 compared to conventional (*p* < 0.001) but not organic (*p* = 0.052) crops. According to further post hoc analyses, conventional oats also contained significantly higher levels of ENB than organic crops (*p* = 0.0022). No significant differences were found for the other detected toxins, occurring at similar levels in oats from each of the three production systems.

## 3. Discussion

### Natural Co-Occurrence of Mycotoxins in Unprocessed Irish Oat Samples

The extent of the natural occurrence of multiple mycotoxins in unprocessed, domestically produced Irish oats is reported for the first time. The findings presented demonstrate that the quick, easy, cheap, effective, rugged, and safe (QuEChERS)-based sample preparation procedure of De Colli et al. [37] can be readily applied for the extraction and detection of the regulated mycotoxins (aflatoxins, fumonisins, ochratoxin A, zearalenone, T-2, HT-2, and deoxynivalenol), the emerging mycotoxins (beauvericin, alternariol, alternariol-methyl-ether and enniatins), and two masked metabolites (deoxynivalenol-3-glucoside and T-2-glucoside) from oat samples.

Amongst the oats samples assessed, which were representative of Irish oat production in 2015 and 2016, a broad range of mycotoxins were detected. Amongst the 20 different mycotoxins identified, T-2 and HT-2 were the most frequently detected. This is not surprising, as many reports have previously shown that oats produced in northern and north-western Europe are more likely to contain HT-2 and T-2 in comparison to other cereals [16,20,22,23,31,38]. In particular, the UK may represent a solid basis of comparison with Ireland, due to its geographical position and similar weather conditions. Edwards and co-workers conducted extensive research on the occurrence of major *Fusarium* toxins in oats and other cereals in the UK [21,27,29,38,39,40]. In their study published in 2009 Edwards et al. [27] showed that between 2002 and 2005 that combined HT-2 and T-2 toxins were quantifiable in 92% of samples, with mean, median, and maximum concentrations were 570, 213, and 9990 µg kg^−1^, respectively. In comparison, in the presented study we showed a lower prevalence of the combined mycotoxins (51% of all samples), while mean, median, and maximum concentrations were 770, 493, and 4507 µg kg^−1^ (Table 1). The difference of the observed prevalence between the studies may in part be explained by the lower LOQs (10 µg kg^−1^) of the analytical method employed by Edwards (2009) [27]. The combined HT-2 and T-2 concentrations observed in our work were above the EU recommended limits of 1000 µg kg^−1^ for unprocessed oats [10] in 13% of the samples analysed. However, the majority of these samples would likely comply with the current 200 µg kg^−1^ limit recommended for the sum of HT-2 and T-2 in processed oats, considering the reduction of up to 90–95% occurring upon industrial processing of raw oats into oat flakes, as demonstrated in other studies [29,41]. A very high prevalence of T-2 and HT-2 toxins was also observed in 243 unprocessed oat samples collected between 2005 and 2009 from various European mills [22]. T-2 and HT-2 toxins were quantifiable (LOQ = 5 µg kg^−1^) in virtually all of the samples, while the maximum concentrations reported were 269 µg kg^−1^ and 572 µg kg^−1^ and the means 32 µg kg^−1^ and 62 µg kg^−1^, respectively.

In addition to HT-2 and T-2, additional Type A trichothecenes mycotoxins were also detected in the present study. These include NEO, which although structurally related to T-2 toxin is usually omitted in the analytical methods and therefore its presence in food may be largely underestimated. A total of 35 samples (17%) contained detectable levels of NEO in our study, at concentrations ranging from 10.5 to 55 µg kg^−1^. Similarly, the mono-glycosylated conjugate of T-2, T2G, was also detected. Previously, Busman et al. [42] have found glucoside derivatives of T-2 and HT-2 in wheat and oat kernels inoculated with *F. sporotrichiodes*, suggesting that they may also be present in naturally contaminated cereals. Plant-modified T-2 and HT-2 toxins were subsequently reported in naturally contaminated wheat and oats for the first time by Lattanzio et al. [43]. The European Commission have recommended that Member States monitor for the presence of T-2, HT-2, and their masked analogues, in particular the mono- and di-glycosylated conjugates of T-2 and HT-2 toxin [10]. However, reports on their natural occurrence in cereal grains are limited [17,44], probably due to the lack of commercially available standards. In our survey, T2G was quantified in 39 samples (19%), with a maximum concentration of 997 µg kg^−1^. This indicates that T2G may significantly contribute to total toxicity, particularly if it is converted to its original free form upon ingestion by humans or animals, as suggested by Berthiller et al. [13].

In line with the findings reported by Edwards et al. in UK [27], DON and its masked derivative D3G did not appear to be highly frequent in Irish oats. D3G occurred in only two samples (1%), with the highest concentration measured at 115 µg kg^−1^. Its corresponding free form, DON, was merely twice as prevalent and no samples exceeded the current maximum permitted limits of 1750 µg kg^−1^ for unprocessed oats. The differences in prevalence of HT-2, T-2 and T2G to that of DON and D3G is most likely reflective of dominant *Fusarium* species infecting Irish oats. Though various *Fusarium* spp. are reported to produce type A trichothecenes, more recent publications have begun to focus on *F. langsethiae* as a significant producer in the field [16,45,46]. The contributions of this species in the production of T-2 and HT-2 in Irish oats has not yet been verified, although given the dominance of both mycotoxins further investigations are warranted.

OTA and ZEN were the only other legislated mycotoxins detected in this work, occurring in eight (4%) and two (1%) samples, respectively. The presence of OTA has been reported in raw oats and other cereals [26,47,48], breakfast products [18,19], and infant foods [49]. In particular, Kolakowski et al. carried out an extensive investigation of almost 7000 cereal-based, fruit-based, and soy-based food samples collected between 2009 and 2014 in Canada. Almost half (47%) of the 388 oat based samples were found to contain quantifiable levels (>0.5 µg kg^−1^) of OTA, while ten samples exceeded the current EU permitted limits and the maximum concentration determined was 21.2 µg kg^−1^. In comparison, where OTA was detected in the current study it was found at similar levels, although its low detection levels should be noted (Table 1). In addition, our findings showed that all the samples containing OTA were also found to contain MPA, suggesting a fungal infection caused by genus capable of producing both OTA and MPA, such as *Penicillium*. Lund and Frisvad (2003) [50] reported that *Penicillium verrucosum* was the most predominant OTA producer in barley and wheat grown in Europe (Ireland was not included in the sampling) during harvesting, drying, and storage, where an infestation of more than 7% resulted to OTA contamination. It is a very competitive species, able to dominate in stored grains under favourable conditions of environmental water availability, temperature, and water activity which is reflective of the grains moisture content, with increasing levels of each leading to OTA production [51]. The moisture content at the time of milling was recorded for 85 of the 208 samples included in this study. Of these, those with detectable levels of OTA and MPA had a mean moisture content of 19.1% (17.8 to 23.5%), while samples without these two mycotoxins contained an average 16.7% (14.4 to 22.4%) moisture. In addition, it was observed that as moisture content increased, the concentrations of MPA increased accordingly (*r* = 0.5, *p* = 0.011). However, it should be highlighted that these unprocessed oat samples were milled and stored upon sampling, and it can be expected that, as harvested oats undergo the normal post-harvest drying processes in preparation for storage prior to milling for human consumption, the conditions leading to OTA and MPA production will be greatly reduced.

Only two oat samples contained ZEN at concentrations higher than the LOQ (20 µg kg^−1^), and both samples did not exceed the permitted level of 100 µg kg^−1^. Much higher maximum concentrations were found in oat samples collected from Sweden and Norway, both reportedly containing ZEN around 2300 µg kg^−1^, which may reflect on different conditions or fungal species found in each country [31,32].

Enniatins, particularly ENA, ENA1, ENB, and ENB1, are among the most commonly occurring, emerging mycotoxins in agricultural products, especially the small grain cereals, where several *Fusarium* species including *F. avenaceum*, *F. acuminatum*, *F. poae*, *F. sporotrichioides*, *F. tricinctum*, *F. torulosum*, *F. venenatum*, *F. compactum*, *F. proliferatum*, *F. subglutinans*, and *F. temperatum* have been reported as producers [12]. Currently, no regulations have been set by the EU for maximum permissible levels of enniatins (and other emerging mycotoxins) in cereals, due to the limited data available on their toxicity. However, a number of surveys have been conducted in the last 10–15 years, mostly in northern European countries. In four different studies on oats and other cereals carried out in as many Scandinavian countries (Norway, *n* = 73; Sweden, *n* = 93; Denmark, *n* = 11; Finland, *n* = 153), enniatins were detected in all (100%) the samples analysed [16,31,33,52]. In a more recent study, a total of 289 oat samples were collected in Norway over a six-year period from 2004 to 2009. Enniatins were detected in up to 95% of the samples, depending on the compound (LOQs ranging from 3 to 5.4 µg kg^−1^) and the year of sampling, while the highest concentrations determined were 6800 µg kg^−1^ (ENB) and 6200 µg kg^−1^ (ENB1) [32]. The number of positive detections in our study, from high to low, was ENB>ENB1>ENA1 (Table 1), in agreement with reports elsewhere [32,33]. Although recent surveys showed relatively low levels of contamination (i.e., <100 µg kg^−1^), enniatins have also been reported at concentrations exceeding several ppm [32]. The median concentrations measured in unprocessed Irish oats ranged from 142 (ENA1) to 226 (ENB1) µg kg^−1^, while the highest concentration was measured for ENB at 1517 µg kg^−1^ (Table 1). However, there are still limited data available on the prevalence and contamination levels of enniatins in particular when compared to the ‘traditional’ *Fusarium* toxins.

Other compounds that are commonly referred as “emerging” mycotoxins include beauvericin (BEA), moniliformin (MON), sterigmatocystin (STER), fusaproliferin, fusaric acid, culmorin, emodin, mycophenolic acid, and other *Alternaria* toxins such as tenuazonic acid, alternariol (ALTOH), alternariol monomethyl ether (AME), and their metabolites [53,54]. BEA resembles enniatins structurally, and it is often found co-occurring with them, although at lower concentrations and prevalence [54]. STER, a precursor of AFB1, is classified as a Group 2B carcinogen by the International Agency for Research on Cancer and it is recently gaining consideration among the scientific community [53]. ALTOH, AME and their related molecules may reportedly be found in different grains, particularly sorghum, if a high moisture content is present in the field [55]. With exception of MPA, these toxins never exceeded 5% prevalence in the samples assessed. However, relatively high concentrations of STER (458 µg kg^−1^), BEA (150 µg kg^−1^) and MPA (7362 µg kg^−1^) were measured in some cases, again highlighting the continued need to monitor emerging and modified mycotoxins in order to collect sufficient data on their occurrence and enable food safety authorities to establish whether maximum permitted levels are required.

Levels of mycotoxin contamination in oats can vary greatly across years, depending on factors such as climate, variety, sowing date, and pre-crop rotation [23]. However, the precise contributions of each factor are unclear. A number of authors have tried to address the issue in several studies over the years [14,21,24,27,29,32,34,39,56,57,58,59,60]. One of the more prominent research groups in this literature, Edwards et al. [57] utilised a large U.K. dataset spanning the seasons 2002–2008, finding crop type (winter or spring) and variety to impact levels of contamination, with significantly higher HT-2 and T-2 concentrations detected in winter-sown oats compared to spring. However, as the sowing date may be considered an important risk factor separate from genetic differences between plant varieties, the authors highlighted that further studies would require the same varieties to be sown in replicated field experiments at different sowing dates, such as conducted by Orlando et al. for barley [61]. In the present work, the prevalence of mycotoxins was highly variable across the oats sown in winter and spring. Although the concentrations measured were comparable for most mycotoxins, the spring 2015 samples contained significantly higher levels of the combined HT-2 and T-2 compared to both winter 2015 and spring 2016. Similarly, winter 2015 oats appeared to contain higher amounts of HT-2 and T-2 than winter 2016. As shown in Supplementary Figure S1, May 2015 was characterised by relatively high rainfall, and both April and June of the same year were much drier in comparison, while November and December were again exceptionally rainy compared to October [62]. Interestingly, the following year recorded an opposite scenario, with generally drier weather conditions from the summer to the end of the year. As booting-panicle emergence for Irish winter sown oat crops is generally in mid-late May and for spring sown crops in mid-late June these differences are supported by modelling by Xu et al. [59], who reported that the effects of environmental conditions on HT-2 and T-2 accumulation in predominantly winter sown field oat grains in the UK was positively related to wet conditions during early May and dry conditions from late May, which likely corresponds to the period just prior to anthesis, until harvest. However, given the limited time frame investigated, multi-annual surveys are needed to clarify these aspects.

A tendency toward generally higher mycotoxin content was observed in the conventionally grown oats compared to organic and gluten free crops, though the trend was not always statistically significant, such was the case for HT-2, T-2, T2-3OH, T2G, and ENB1; though ENB was measured at levels significantly higher (Figure 4 and Figure 5). Several studies have discussed whether conventional or organic farming systems have a significant impact on the mycotoxin contamination of oats (and other cereals), but findings are often unclear [14,20,27,30,34,48,63,64,65,66,67] and when combined suggest that weather conditions, locations, crop rotation, and other agronomic factors are more important for the development of the major mycotoxins than the specific type of farming (conventional vs. organic). Significantly, the vast majority of the published surveys have omitted the emerging and masked mycotoxins, and gluten free cereals were not investigated. In comparison to the gluten free samples in our study, the conventionally produced oats contained significantly higher concentrations of HT-2 (Figure 5), while MPA, OTA, OTB, and STER showed a lower detection rate (Figure 4). As the gluten free samples will have been produced under strict adherence to crop rotation with a previous non-cereal crop, the difference in HT-2 concentrations is likely to reflect differences in *Fusarium* infections resulting from crop rotation. Edwards [27] has previously suggested that the differences in mycotoxin contamination between organic and conventional may in part be due to rotations with non-cereals. For the latter four mycotoxins with elevated levels in the gluten free samples, as commercial gluten free oats are immediately dried, milled, and further processed after harvest, contamination with these mycotoxins is likely to be significantly reduced as described above.

## 4. Conclusions

The co-occurrence of multiple mycotoxins, including the emerging and masked mycotoxins, has been reported in Irish oats for the first time. A range of 20 out of 42 different toxins were measured, and T-2 and HT-2 were the most frequently detected in the samples analysed. The masked mycotoxins D3G and T2G were quantified in 1% and 19% of the samples, respectively. DON, OTA, and ZEN were the only legislated mycotoxins detected. The outcome of this work indicated that Irish unprocessed oats are quite safe with regard to the current regulated mycotoxins, although some of the measured concentrations of T-2 and HT-2 toxins may represent the possibility of greater compliance issues in the future, should climatic and other influential factors further favour their production in the field. Nevertheless, it should be highlighted that standard industrial processes, such as de-hulling, have been proven effective in reducing T-2 and HT-2 concentrations by up to 90–95%. A number of mycotoxins of increasing toxicological interest were also detected, including ALTOH, BEA, ENA, ENA1, ENB, ENB1, MPA, NEO, NIV, and STER. This, once again, highlights the need to test for multiple compounds other than the traditionally screened toxins. The year of sampling had a minor influence on the mycotoxin content of Irish oats, while sowing season and farming system seemed to have a higher impact on the production of certain mycotoxins. Significantly lower concentrations of HT-2 were quantified in oats from gluten free farming. However, the prevalence and concentrations of mycotoxins are influenced by multiple, interactive factors, including climate, plant variety, sowing season, and crop rotation. Systematic comparisons using controlled experimental field trials and multi-annual surveys are needed to clarify these aspects. In addition, the predominant fungal species in oat producing fields in Ireland should be investigated, especially in light of the foreseen climate change that may result in the production of unexpected mycotoxins due to migration of toxigenic fungi from more temperate latitudes.

## 5. Materials and Methods

### 5.1. Chemical Reagents and Apparatus

All the chemical reagents were of analytical grade. Ultra-pure water (18.2 MΩ-cm) was generated in-house using a Millipore water purification system (Millipore, Cork, Ireland). Acetonitrile (ACN), methanol (MeOH), and isopropyl alcohol (IPA) were supplied by Romil Ltd. (Cambridge, UK). Ammonium acetate and dimethyl sulfoxide (DMSO) were supplied by Sigma-Aldrich (Dublin, Ireland). Acetic acid 100% (CH_3_COOH, LC-MS grade) was provided by Merck (Darmstadt, Germany). Anhydrous magnesium sulphate (MgSO_4_) and sodium chloride (NaCl) were sourced from United Chemical Technologies (Wexford, Ireland) and Applichem (Darmstadt, Germany), respectively.

An ME36S microbalance and an A200S digital electronic analytical balance, both from Sartorius (Dublin, Ireland), were used for weighing the standard materials and solvents. A Rotanta 460R centrifuge (Hettich, Kirchlengern, Germany), Gerhardt shaker (Interplant, Dublin, Ireland), Merris Minimix Vibrational Shaker (Merris Engineering, Galway, Ireland), TopMix multi-vortexer (Fisher Scientific, Dublin, Ireland), and a TurboVap LV evaporator from Biotage (Uppsala, Sweden) were used for sample preparation. A Tecator Cyclotec 1093 mill (FossElectric DK3400 Hilleroed, Denmark) fitted with a 1 mm sieve was used for milling the samples prior to sample extraction. Two different sizes of polypropylene tubes (15 mL and 50 mL) were obtained from Sarstedt Ltd. (Wexford, Ireland). Ceramic homogeniser pellets (15 mL tubes, part number 5982-9312) were purchased from Agilent Technologies Ltd. (Cork, Ireland), and syringeless mini-uniprep PTFE filter devices were sourced from Whatman plc (Maidstone, UK).

### 5.2. Sample Collection and Experimental Design

A total of 208 unprocessed oat samples (ca. 2 kg each) were collected from five Irish processors over 2015 (*n* = 90) and 2016 (*n* = 118). While commercial crop harvests normally undergo controlled drying and storage procedures, these raw and unprocessed samples were transported to a central collection facility and milled using a Tecator Cyclotec 1093 mill fitted with a 1 mm sieve, then stored at −20 °C prior to mycotoxin analysis. The samples were analysed within few weeks/months upon collection and storage.

The sample set comprised of 77 crops grown under a “gluten free” system, 19 organically grown crops, and the remaining 112 samples derived from crops grown using conventional farming inputs (e.g., fertiliser, herbicides, insecticides, and growth regulators). In the case of the organically grown crops and as per standard regimes, no synthetic plant protection products were applied while organic fertilisers were used. Though *Avena sativa* does not produce gluten, modern crop rotation practices can potentially lead to a significant risk of gluten contamination in oat crops from previously grown cereal crops in the same field. Hence, a specific “gluten free” system indicates care has been taken to include non-cereal crops in the rotation prior to planting the oat crop, which then receives all the aforementioned conventional farming inputs.

The samples were grown in several farms across Ireland and collected from two sowing seasons each year: spring (2015, *n* = 51; 2016, *n* = 35) and winter (2015, *n* = 39; 2016, *n* = 74). Nine samples were excluded from inter-seasonal analyses (*n* = 199) because their sowing season was not recorded. In Ireland oats are grown as either spring or winter crops, with spring oats generally sown between late March to early April and harvested in late August, while winter oats are sown from October to early November and harvested in late July or early August of the following year. For example, the “winter 2015” crop was sown before the end of 2014, and harvested in 2015. Winter sown crops will tend to be booting from mid-late May, whilst spring sown crops will be mid-late June.

### 5.3. Sample Preparation and UHPLC-MS/MS Analysis

The sample preparation procedure and UHPLC-MS/MS analysis were performed as described by De Colli et al. [31]. Briefly, a QuEChERS-based protocol was employed for isolating mycotoxins from oat samples. A 10 mL volume of 1% aqueous acetic acid and 10 mL of ACN were sequentially added to 50 mL polypropylene centrifuge tubes containing 2 g of milled oat samples, which were shaken at 200 oscillations min^−1^ on a horizontal shaker for 5 min after each addition. Following partitioning by MgSO_4_ and NaCl, samples were vigorously shaken using a Minimix Vibrational Shaker and then centrifuged. For the analysis of ROQE, ENB1, ENB, BEA, ENA, and ENA1, 250 μL of the organic phase was filtered and injected directly. For the remaining analytes, a 2.5 mL aliquot of the supernatant was evaporated to dryness and reconstituted in 200 μL of H_2_O:MeOH:DMSO (80:10:10, *v*/*v*/*v*) prior to filtration and injection onto the UHPLC-MS/MS. Oat samples that did not contain any detectable level of mycotoxins were used for preparing the matrix-matched calibration curves. After fortification, the samples were allowed to stand for at least 15 min, then extracted and analysed using the same analytical method. The sample analysis was performed using a Waters Acquity UPLC system coupled to a Waters Quattro Premier XE triple quadrupole mass spectrometer (Milford MA, USA), interfaced with an ESI ionisation probe. Chromatographic separation was carried out on a Waters Acquity BEH C_18_ (2.1 × 100 mm, particle size 1.7 μm) column, fitted with an in-line filter with 0.2 μm pore size, and maintained at 40 °C. A binary gradient separation was employed comprising of mobile phase A: 5 mM ammonium acetate in H_2_O:MeOH (90:10, *v*/*v*) and mobile phase B: 0.2% CH_3_COOH and 5 mM ammonium acetate in MeOH at a flow rate of 0.4 mL min^−1^.

### 5.4. Statistical Analysis

The software R 4.0.2 (R Foundation for Statistical Computing, Vienna, Austria) together with the ggpubr (v0.4.0) and FSA (v0.8.3) packages were used for statistical analysis [68,69,70]. Group comparisons (e.g., inter-season; inter-treatment; et cetera) of observed concentrations were performed by Kruskal–Wallis H test; null hypotheses were rejected, and association considered significant at (*) *p* < 0.05; (**) *p* < 0.01; (***) *p* < 0.001. Where significant differences were identified, post hoc pairwise comparisons were performed by Dunn’s rank-sum test using the Benjamini–Hochberg procedure for correcting family-wise error rate; associations were considered significant at the same *p*-value cut-offs. Where significant differences were identified between groups, post hoc power calculations were performed to ensure reliability; statistical power was calculated above 99% in all relevant tests. In the boxplot graphs, the upper whisker extends from the box to the largest value no further than 1.5 × IQR above the third quartile (Q3), where IQR is the inter-quartile range, or distance between the first and third quartiles. The lower whisker extends from the box to the smallest value at most 1.5 × IQR lower than first quartile (Q1). Data beyond the ends of the whiskers are called outlying points and are plotted individually.

## Figures and Tables

**Figure 2:**
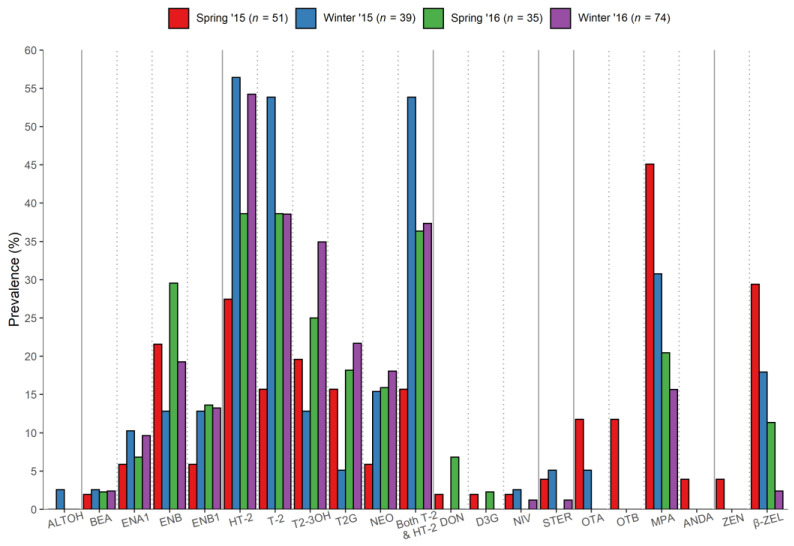
Prevalence (%) of the mycotoxins detected in both spring and winter oat samples over two consecutive years (2015–2016).

**Table 1:** Overview of the 20 detected mycotoxins (µg kg^−1^) in 208 unprocessed Irish oat samples collected over two years (2015–2016).

Mycotoxin	%*p*/*n* (*p*)	Mean (Median) (µg kg^−1^)	Min–Max (µg kg^−1^)	LOQ (µg kg^−1^)
ALTOH	0.5 (1)	41.7 (41.7)	41.7–41.7	20.0
ANDA	1.0 (2)	723 (723)	720–726	50
BEA	2.9 (6)	47 (26.6)	25.6–150	25.0
D3G	1.0 (2)	71 (71)	26.8–115	12.5
DON	2.4 (5)	212 (211)	131–330	100
ENA1	8.7 (18)	142 (125)	63–347	50
ENB	23.6 (49)	304 (202)	100–1517	100
ENB1	12.5 (26)	300 (226)	102–1021	100
HT-2	51.4 (107)	514 (291)	53– 3405	50
MPA	27.9 (58)	1138 (377)	52–7362	50
NEO	16.8 (35)	21.9 (17.4)	10.5–55	10.0
NIV	1.9 (4)	193 (161)	114–338	100
OTA	3.8 (8)	10.2 (9.4)	2.9–19.7	2.5
OTB	2.9 (6)	4.1 (4.3)	1.9–5.9	1.0
STER	2.4 (5)	234 (114)	89–458	50
T-2	41.3 (86)	256 (202)	55–1,102	50
T2-3OH	28.4 (59)	55 (45.3)	12.9–159	10.0
T2G	18.8 (39)	153 (98)	26.0–997	25.0
β-ZEL	13.9 (29)	617 (432)	38.1–2498	20.0
ZEN	1.0 (2)	29.6 (29.6)	29.2–29.9	20.0

Calculated statistics describe positive detections (*p*) only; *p*, contaminated samples (%); LOQ, limit of quantification (lowest point of the calibration curve); Min, minimum values; Max, maximum values.

## Data Availability

The data presented in this study are available within the article or supplementary material.

## Supplementary Materials

The following are available online at https://www.mdpi.com/2072-6651/13/3/188/s1, Figure S1: Comparison of the monthly average rainfall (mm, upper) and temperature (°C, lower) across the island of Ireland for the years 2015 and 2016; Table S1: List of mycotoxins that were not detected and corresponding limit of quantification (LOQs).
