# Supplementary material for: Natural Co-Occurrence of Multiple Mycotoxins in Unprocessed Oats Grown in Ireland with Various Production Systems
Source: Toxins (Basel). 2021 Mar 4;13(3):188. doi: 10.3390/toxins13030188 (PMC7998419; doi:10.3390/toxins13030188)
Supplement: Supplementary file 1 [file toxins-13-00188-s001.pdf]

Supplementary Materials: Natural Co-Occurrence of Multiple Mycotoxins in Unprocessed Oats Grown in Ireland with Various Production Systems

Lorenzo De Colli, Karl de Ruyck, Mohamed F. Abdallah, John Finnan, Ewen Mullins, Stephen Kildea, John Spink, Christopher Elliott and Martin Danaher

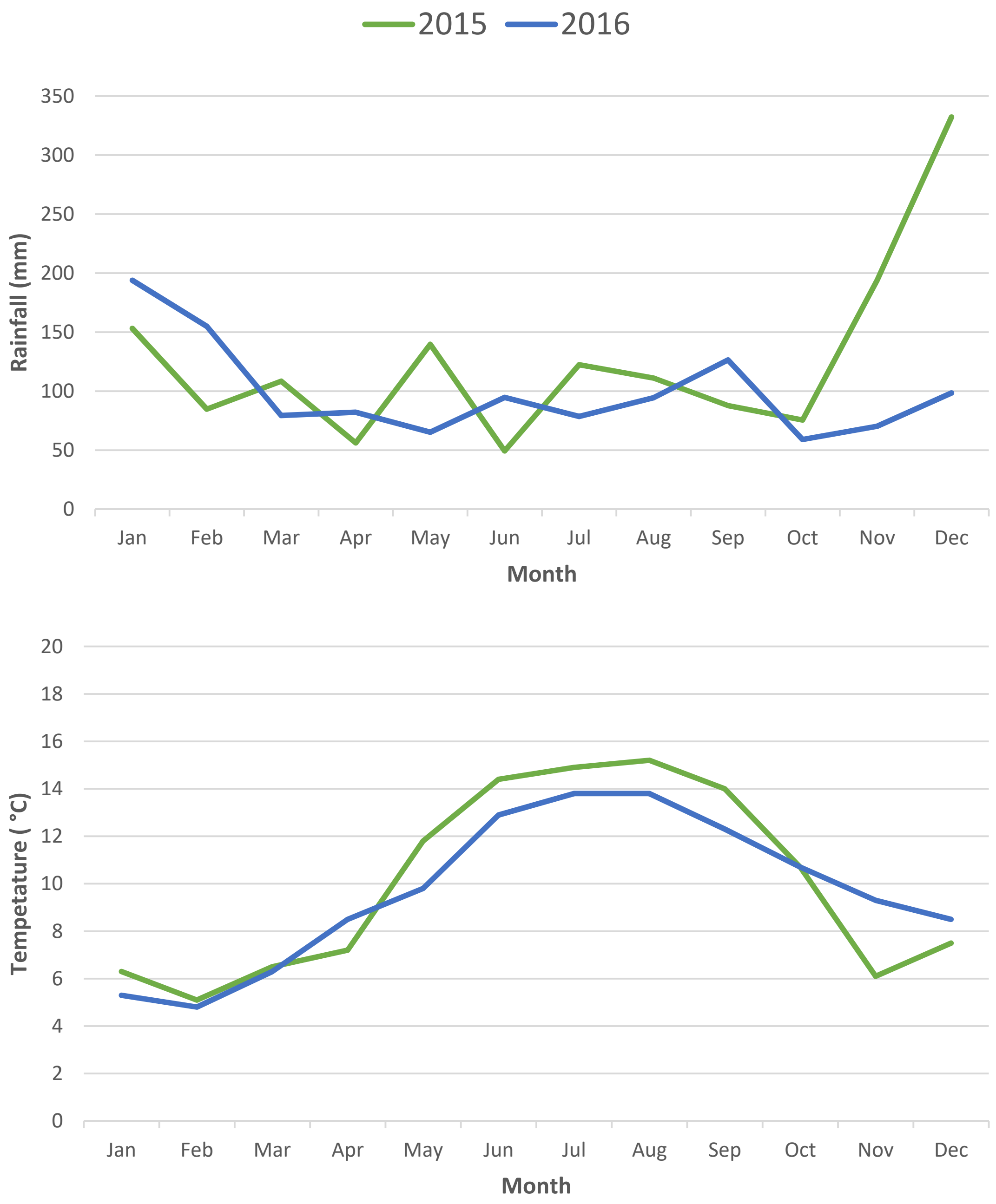

Figure S1. Comparison of the monthly average rainfall (mm, upper) and temperature (°C, lower) across the island of Ireland for the years 2015 and 2016.

Table S1. List of mycotoxins that were not detected and corresponding limit of quantification (LOQs).

**Undetected Mycotoxins**	**LOQ (μg kg^{-1})**
PAT	10.0
FUS-X	25.0
DOM-1	20.0
3ADON	100
15ADON	100
AFG2	0.50
AFG1	0.50
AFB2	0.50
AFB1	0.50
GLIO	50
DAS	10.0
WORT	50
TEN	20.0
FB1	20.0
ROQC	50
α-ZEL	20.0
TERRB	25.0
FB3	10.0
AME	20.0
FB2	20.0
ROQE[c]	50
ENA[c]	50
